# Development of a laser-based heating system for *in situ* synchrotron-based X-ray tomographic microscopy

**DOI:** 10.1107/S0909049512003287

**Published:** 2012-03-16

**Authors:** Julie L. Fife, Michel Rappaz, Mattia Pistone, Tine Celcer, Gordan Mikuljan, Marco Stampanoni

**Affiliations:** a Laboratory for Synchrotron Radiation, Swiss Light Source, Paul Scherrer Institut, Villigen, Switzerland; b Computational Materials Laboratory, Ecole Polytechnique Federale de Lausanne, Lausanne, Switzerland; c Institute for Geochemistry and Petrology, Swiss Federal Institute of Technology of Zurich, Zurich, Switzerland; d The Centre of Excellence for Biosensors, Instrumentation and Process Control, Solkan, Slovenia; e Institute for Biomedical Engineering, Swiss Federal Institute of Technology and University of Zurich, Zurich, Switzerland

**Keywords:** *in situ* X-ray tomographic microscopy, ultra-fast imaging, diode lasers, metals solidification, volcanic processes

## Abstract

Understanding the formation of materials at elevated temperatures is critical for determining their final properties. Synchrotron-based X-ray tomographic microscopy is an ideal technique for studying such processes because high spatial and temporal resolutions are easily achieved and the technique is non-destructive, meaning additional analyses can take place after data collection. To exploit the state-of-the-art capabilities at the tomographic microscopy and coherent radiology experiments (TOMCAT) beamline of the Swiss Light Source, a general-use moderate-to-high-temperature furnace has been developed. Powered by two diode lasers, it provides controlled localized heating, from 673 to 1973 K, to examine many materials systems and their dynamics in real time. The system can also be operated in various thermal modalities. For example, near-isothermal conditions at a given sample location can be achieved with a prescribed time-dependent temperature. This mode is typically used to study isothermal phase transformations; for example, the formation of equiaxed grains in metallic systems or to nucleate and grow bubble foams in silicate melts under conditions that simulate volcanic processes. In another mode, the power of the laser can be fixed and the specimen moved at a constant speed in a user-defined thermal gradient. This is similar to Bridgman solidification, where the thermal gradient and cooling rate control the microstructure formation. This paper details the experimental set-up and provides multiple proofs-of-concept that illustrate the versatility of using this laser-based heating system to explore, *in situ*, many elevated-temperature phenomena in a variety of materials.

## Abstract 2

A laser-based heating system has been developed at the TOMCAT beamline of the Swiss Light Source for *in situ* observations of moderate-to-high-temperature applications of materials.

## 1. Introduction

Non-destructive synchrotron-based X-ray tomographic microscopy is ideal for studying opaque materials. This is because the high flux of hard X-rays at a third-generation source reveals volumetric microstructural information in a matter of minutes. A series of projections (*i.e.* X-ray radiographs) are captured on an imaging detector over a set of angular positions and the resulting information, traditionally based on X-ray absorption of the elements in the material, reveals the microstructure. Couple this technique with a newly developed ultra-fast data acquisition endstation, where a full three-dimensional data set of several hundred projections over 180° can be captured in less than 1 s, and one can begin to explore and characterize many dynamic systems in real time (Mokso *et al.*, 2010 ▶). Real time, in this case, means that the speed of the scan is quicker than the dynamics of formation and/or growth in the microstructure and is thus adequate to resolve these changes. Such an endstation, with unprecedented spatial and temporal resolution, is located on the tomographic microscopy and coherent radiology experiments (TOMCAT) beamline of the Swiss Light Source (SLS) (Stampanoni *et al.*, 2006 ▶). The spatial resolution, typically 1–10 µm, and field of view, ranging from 1 to 22 mm, found at TOMCAT can be tailored for various materials and applications. Contrast can be achieved through multiple methodologies: from standard absorption, typically used in metal and composite systems, to propagation-based and grating-based phase contrast, predominantly used for biological and other traditionally low-contrast materials. Finally, the efficient image-processing pipeline (Hintermueller *et al.*, 2010 ▶; Marone *et al.*, 2010 ▶) provides a full three-dimensional tomographic reconstruction within minutes, making visualization close to real time in most situations.

Owing to these significant advancements in data acquisition, understanding the dynamics of materials systems is becoming a reality. Often, elevated temperatures are necessary to achieve such studies and, thus, incorporating a furnace system into a beamline set-up is a significant step forward. Previous work at synchrotrons and spallation sources (Landron *et al.*, 2000 ▶; Bellet *et al.*, 2003 ▶; Li *et al.*, 2006 ▶; Mathiesen *et al.*, 2002 ▶; Buffiere *et al.*, 2010 ▶; Billia *et al.*, 2010 ▶) have shown that various elevated-temperature apparatuses can be designed and tailored to achieve successful results in specific applications. While such systems work well for these well defined problems, there remains a need to achieve multiple heating modalities in a compact set-up. This broadens the applicability of the set-up to general beamline users interested in elevated-temperature experiments while minimizing limitations in experimental design. Further, multiple heating modalities create an opportunity to study a wider variety of elevated-temperature phenomena, from defect formation and microstructure evolution under isothermal conditions to branching mechanisms and competitive growth in thermal temperature gradients. Using lasers as the source of heat is very attractive because they provide the versatility to meet the thermal criteria for such a furnace set-up. With small changes to the set-up, the furnace can switch between near-isothermal conditions to linear temperature gradients in broad temperature ranges, and the furnace can accommodate a large spectrum of sample sizes and materials because there are minimal sample size constraints. Further, because there are no bulky heating elements or additional holders, the X-ray detector can remain close to the sample, which reduces edge-enhanced artifacts in the resulting images. In situations where edge-enhancement is attractive for purposes of creating contrast, the furnace set-up is also compatible.

This paper details the design and implementation of a furnace powered by two class 4 diode lasers on the TOMCAT beamline of the SLS. We also present results coupling the laser system to the ultra-fast endstation recently commissioned at TOMCAT to examine two elevated-temperature phenomena: solidifying metal ingots and simulating volcanic processes in geologically derived materials. Examples using both standard data acquisition and ultra-fast data acquisition with the new laser-powered furnace are shown.

## 2. Experimental method

### 2.1. Laser design

We have developed a furnace that incorporates two near-infrared (IR) diode lasers, operating at a wavelength of 980 nm and with a power output of 150 W each, manufactured by Apollo Instruments (Irvine, CA, USA). Diode lasers have advantages over other laser systems that make them attractive for use on a beamline set-up. These include high efficiency, which reduces energy consumption, low cost and low maintenance, guaranteeing at least 12000 h of working life. Each laser has a rectangular spot 0.2 × 1 mm in size at a working distance of 40 mm. To achieve furnace-like heating, the lasers are positioned approximately 180° apart with the 1 mm dimension of the spot being horizontal, thus reducing radial thermal gradients in the sample. For safety purposes, the lasers are inclined at an angle of 20° with respect to horizontal so that they are not powered directly into each other. Additionally, two beam blockers are mounted on the complementary decline so that the portion of the beam that does not fall on the specimen is absorbed by these stoppers. This X-shaped set-up is then attached to three linear stages that control the positioning of the lasers before and during data acquisition. These linear stages provide 25 mm of movement in each direction creating flexibility in the set-up for numerous sample sizes and shapes. Further, the sample can move synchronously with the laser both perpendicular to the beam path and vertically such that the heating location remains constant during data acquisition.

In general, the laser system operates under ambient conditions, but is not limited to such an environment. It accommodates samples 1–5 mm in diameter and up to 3 cm in height, which can be extended to larger-diameter samples if necessary. The sample must be contained in an X-ray-transparent sample holder, such as boron nitride, alumina or quartz, and this material is specified by the user or the particular application. Two visible spot lasers (class 2) are included in the set-up to determine the location of the lasers on the sample prior to heating. These alignment spots are critical for assuring that the lasers are centred on the sides of the sample and that the precise user-defined position on the sample is receiving the laser power.

The temperature is read from a non-contact IR temperature measurement device, or pyrometer, developed by Optris (Berlin, Germany). The pyrometer is a class 2 laser that filters out IR and near-IR wavelengths such that the class 4 lasers are invisible to the temperature reading. It can be tailored to the emissivity of the containment vessel or sample material for precise temperature measurement, to within 0.1 K. The pyrometer is also mounted on three manual micrometre stages that allow the user to place the pyrometer spot anywhere along the sample height, either at the same location on the sample as the application of the laser power or not. Further, the pyrometer is mounted on the same vertical stage as the lasers such that the temperature measurement location changes relative to the laser location as samples or specifications change.

The custom-made control system is a standard PID controller that adapts the power of the lasers to a user-specified temperature profile in real time based on readings from the pyrometer. The set-up allows for linear temperature ramps and descents in user-defined intervals as well as isothermal holding of the temperature for up to several hours. Further, the lasers can be operated in manual or automatic modes. In manual mode the user varies the laser power to create the temperature changes he wishes to achieve. In automatic mode the user specifies the material type and the ramp profiles for his experiment. The PID parameters have been tailored to the emissivities of the various containment vessels recommended for use, and, thus, by specifying the line-of-sight material, the readout from the pyrometer is adjusted. This ensures that the temperatures are read accurately for the user-specified system and the profile typically follows what is specified to within 2–3 K.

The control system was designed and implemented in EPICS (Experimental Physics Industrial Control System) (Dalesio *et al.*, 1994 ▶), which is the standard communication framework for beamline and external equipment control at the SLS as well as many other synchrotrons and large research facilities around the world. This implementation ensures that future enhancements of the beamline will work seamlessly with the laser system, and modifications to the current control system would be easy to implement.

**Figure 1:**
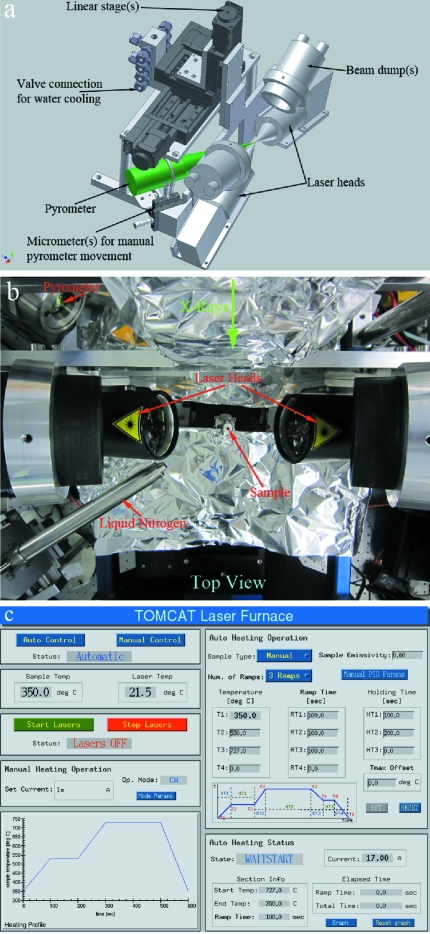
(*a*) Three-dimensional schematic and (*b*) photograph of the laser system mounted on the TOMCAT beamline (X02DA port of the SLS). For scale, the ‘sample’ shown in (*b*) is 2 mm in diameter. (*c*) The laser control panel in EPICS.

A schematic of the proposed set-up, a photograph of the implemented design, and the EPICS control panel are shown in Fig. 1 ▶.

### 2.2. Sample preparation

Binary metal microstructures are commonly analyzed using synchrotron-based X-ray tomographic microscopy, especially in the case of Al alloys (*e.g.* Salvo *et al.*, 2010 ▶). Al-20 wt% Cu was chosen in this case owing to the significant absorption contrast between the two materials. The Al-rich dendrites appear darker (in reverse contrast) than the Al–Cu eutectic/liquid because they absorb fewer X-rays since they are less dense. Typical dendritic features, such as tip radius and primary and secondary arm spacings, are in the 5–500 µm range and thus commonly observed using tomographic microscopy techniques.

Geologically relevant materials are also readily observed at TOMCAT, and with the addition of elevated-temperature capabilities there is a specific interest in simulating volcanic processes on very small scales. These studies would give valuable insight into lava flow and how trapped fluids and gases are released when heat is rapidly applied to the closed system. They are also imperative for eruptive dynamic simulations. The samples can be real geological materials taken from active volcanic areas around the world, or they can be created in a laboratory with similar compositions and specifications to real counterparts. In these experiments a sample of phonolitic obsidian was obtained from the lava flow of Las Canadas Caldera (Tenerife Island, Spain). It was originally composed of pure glass containing 0.23 wt% water and approximately 3 vol% of stretched vesicles.

### 2.3. TOMCAT beamline set-up

Two set-ups available on the TOMCAT beamline are compatible with the new laser-based heating system. The first is the standard absorption set-up, where monochromatic energy is tuned to the sample material and standard microscope objectives are used to magnify the internal structure. A 20 µm LuAG:Ce scintillator converts the X-rays to visible light. The highest flux energy, specifically 21.5 keV, results in the fastest data acquisition; thus, individual projections with 0.37–3.5 µm pixel size can be captured in approximately 60–200 ms.

The second experiment takes advantage of the newly commissioned ultra-fast endstation at TOMCAT (Mokso *et al.*, 2010 ▶). Using polychromatic (white-beam) radiation instead of monochromatic radiation from the 2.9 T super­bending magnet at the SLS increases the flux of photons by two orders of magnitude and creates a continuous spectrum of energies and concomitantly decreases the amount of time the sample is exposed to the X-rays. Such a set-up is ideal for the newly acquired high-speed pco.Dimax CMOS camera, which has the capabilities to acquire and read out individual projections orders of magnitude faster than traditional CCD cameras. Thus, using 5% and 50% filters on the polychromatic beam, we are currently able to acquire an individual projection, with 1–3 µm pixel size, in 1–5 ms. Further, this set-up is limited, mechanically, by the rotation stage, to a minimum acquisition time for a full three-dimensional data set of 0.5 s. Future experiments at TOMCAT (*ca* mid-2012) will incorporate a new rotation stage that will remove this physical limitation.

In preparation for the solidification experiments at TOMCAT, samples 1 mm in diameter and 3–5 mm in height were cut out of larger Al-20 wt% Cu ingots that were previously directionally solidified (Mendoza *et al.*, 2003 ▶). For the geomaterials experiments, samples 2 mm in diameter and 2 mm in height were cut out of larger pieces of the obsidian. Hot-pressed boron nitride (BN) rods provided by Goodfellow Cambridge Ltd (England) were custom manufactured into sample holders that are slightly larger in diameter than the sample sizes, which allows for thermal expansion of each material (for BN, the thermal expansion is 36 × 10^−6^ at 1273 K). BN is relatively ideal for such experiments because of its chemical and thermal stability. It also has a moderate thermal conductivity (15–50 W mK^−1^), providing enhanced heat transfer to the sample, and an upper use temperature of 2773 K.

After machining, the BN holder was inserted into a zirconia rod that was attached to the rotation stage. The heating location was approximately 95 mm above the rotational stage to accommodate the height of the lasers. This also provided space for a stream of liquid nitrogen to be added to the set-up to blow directly on the zirconia rod. This ensured the rotation stage, which has temperature-sensitive micro-mechanical parts, stayed at room temperature.

## 3. Results and discussion

**Figure 2:**
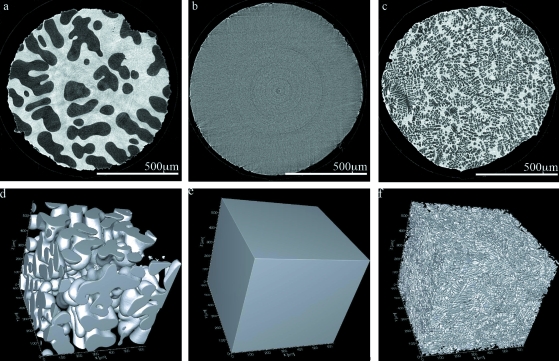
Two-dimensional (*a*, *b*, *c*) and three-dimensional (*d*, *e*, *f*) representations of an Al-20 wt% Cu binary metal microstructure solidifying using the laser-based furnace. Panels (*a*, *d*) show the original unheated microstructure, (*b*, *e*) show the sample just below 923 K, and (*c*, *f*) show the final structure as-solidified. The Al-rich dendrites are shown in (*a*, *c*, *d*, *f*) and the eutectic is transparent. The fully liquid structure is shown in (*b*, *e*) indicating there are no dendrites at this temperature.

Fig. 2 ▶ shows the first *ex situ* solidification results of an Al-20 wt% Cu sample using the laser-based furnace in the standard tomographic microscopy set-up. The lasers were aligned just above the bottom of the sample holder, which was visible in the live preview of the camera. An energy of 21.5 keV was used along with 10× objective magnification which provided a 1.5 mm field of view. The data was binned to decrease the acquisition time of the scans. This resulted in a 1.5 µm pixel size. 721 projections were captured over 180° in approximately 2.5 min.

Figs. 2(*a*) and 2(*d*) ▶ show, respectively, the two-dimensional and three-dimensional representations of the initial dendritic, or tree-like, microstructure prior to heating. This sample was previously coarsened at 838 K for 10 min (Fife & Voorhees, 2009 ▶), explaining the larger-than-expected initial microstructural features. Figs. 2(*b*) and 2(*e*) ▶ were captured while the temperature of the sample was held just above the liquidus of the alloy, *i.e.* the sample was fully liquid. Then the sample was slowly cooled through the solid–liquid region, at approximately −5 K min^−1^ for 20 min, where dendrites typically grow and coarsen through diffusion-based interactions (Marsh & Glicksman, 1996 ▶) and capillarity effects (Dantzig & Rappaz, 2009 ▶). Figs. 2(*c*) and 2(*f*) ▶ were then captured after the laser power was turned off and the sample was fully solidified. The significant changes observed in the microstructure from (*a*) to (*f*) indicate that the laser-based heating system is suitable for future solidification experiments.

**Figure 3:**
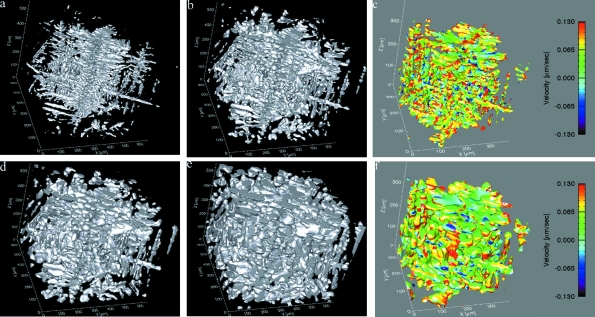
First three-dimensional *in situ* dendrite evolution in Al-20 wt% Cu captured using the laser-heated furnace and the ultra-fast data acquisition at TOMCAT. The Al-rich dendrites are shown in (*a*, *b*, *d*, *e*) while the liquid is transparent. The total evolution time from (*a*) to (*e*) is approximately 200 s. (*c*) and (*f*) show smaller portions of the solid–liquid interface of (*a*) and (*d*), respectively, coloured by the velocity of the moving interface (in µm s^−1^). The velocity was calculated from two experimental microstructures and then displayed back on the original microstructures.

Following this successful *ex situ* experiment of dendrite formation in the standard data acquisition set-up, the first experiments using the ultra-fast endstation were performed (see Fig. 3 ▶). This was an attempt to capture the solidification process in real time. Using polychromatic beam we obtained 17 ms acquisition time with 721 projections captured over 180°; thus, we acquired a full three-dimensional data set in 20 s. The pixel size was 1.1 µm in a 2 mm field of view. The sample was manually cooled from above the eutectic temperature at approximately −0.5 K s^−1^ while full three-dimensional data acquisition was occurring. Figs. 3(*c*) and 3(*f*) ▶ show initial velocity calculations (in µm s^−1^) of the evolving interface, each calculated from two experimental microstructures at a 40 s time interval. It should be noted that, under the current data acquisition specifications and up to the mechanical limit of the maximum speed of the current rotation stage, the effects of centrifugal forces owing to rotation were not observed at any temperatures or in any microstructures.

Al–Cu alloys have weakly anisotropic solid–liquid interfacial energy, typically about 1% (Napolitano *et al.*, 2002 ▶), and additional copper reinforces the growth of the dendrite trunk along the 〈110〉 plane (Friedli, 2011 ▶). The arms then branch at approximately a 90° angle, similar to what is seen in Fig. 3(*a*) ▶. In order to characterize the formation of these dendrites, we would first observe the dendrite tip and primary trunk formation and then capture the initial formation of the secondary arms. Clearly, from Fig. 3(*a*) ▶, the formation of the primary trunk is not separated from the initiation of the secondary arms, and the structure is already relatively well established. Thus, in this case, the initial formation of the dendrites was not captured in real time. It is clear, however, that, as the dynamics decelerate, the temporal resolution is sufficient, see Figs. 3(*d*) and 3(*e*) ▶, to observe the coarsening of dendrites to reveal their final structure. Quantifying this growth begins by calculating the velocity of the solid–liquid interface, as in Figs. 3(*c*) and 3(*f*) ▶. The magnitudes of the velocities are of the order of the average change in the length scale of the system during this time, following *t* ^1/3^ growth kinetics as in the isothermal coarsening of this alloy (Fife & Voorhees, 2009 ▶). Current experiments are focused on modifying the experimental conditions such that increased temporal resolution will capture the initial stages of solidification in real time; this is specifically to see the dendrite tip formation and the initial branching of the secondary arms.

**Figure 4:**
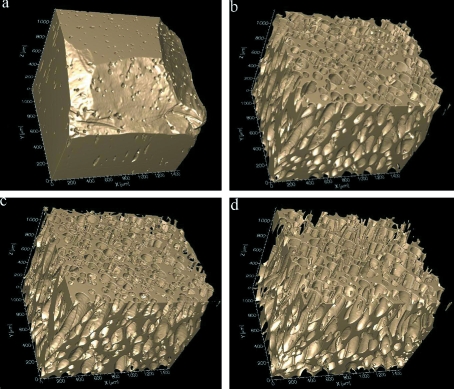
The vesiculation of bubbles, mimicking a small-scale volcanic process, in an obsidian glass sample from Las Canadas Caldera in Spain. The evolution of the melt is shown, while the bubbles are transparent, and the total evolution from (*a*) to (*d*) is 10 s.

Fig. 4 ▶ shows the evolution of Las Canadas Caldera obsidian during rapid heating, at a rate of approximately 15 K s^−1^ between 1073 and 1273 K. Again using the ultra-fast set-up, we obtained 1 ms acquisition times and collected 1001 projections to capture a full three-dimensional data set every 1 s. The pixel size was 2.94 µm and the field of view was 6 mm.

Rapidly heating the obsidian above 1273 K generated bubbles as a by-product of the ex-solution of water from the melt. The water content decreased to approximately 0.10 wt%, measured by Karl–Fischer titration. This, in conjunction with the rapid addition of heat, can be directly linked to the significant decrease in the viscosity of the melt (Giordano *et al.*, 2008 ▶). Additionally, it induced plastic behaviour in the foam which kept all the nucleated bubbles in the melt. As a result, a fourfold expansion of the volume occurred without fracture, predominantly lengthening the sample because the diameter was restricted by the BN holder. Interestingly, the vesicles are elongated, which is best seen in Figs. 4(*b*)–4(*d*) ▶, but their distribution is still relatively polygonal. This is most likely because the viscosity of the melt was still large enough to trap gas in the system and thus the bubbles could not coalesce. More importantly, the elongation of the vesicles is typically considered an indicator of obsidian flow, since they are used as lava flow markers in real systems. Quantification of this information is ongoing and will prove essential for predictive models in eruptive dynamics.

## 4. Conclusions

*In situ* elevated-temperature experiments are now a reality at the TOMCAT beamline of the SLS. We created a compact furnace system using two class 4 diode lasers as the heat source. This system provides near-isothermal and linear temperature gradient capabilities ranging from 673 to 1973 K. It accommodates multiple sample sizes and materials, and it is designed to provide the user with ultimate flexibility in experimental design. The control system gives the user the freedom to change heating profiles as necessary based on real-time insight gained from experimentation. When coupled with the ultra-fast endstation at TOMCAT, dynamic experiments are feasible, as shown with the growth of Al-rich dendrites in an Al–Cu ingot and the mimicking of small-scale volcanic processes in geomaterials. This set-up will prove to be a significant tool for advancing the understanding and characterization of the formation and growth of many materials in real time.
